# Associations of dog and cat ownership with wheezing and asthma in children: Pilot study of the Japan Environment and children's study

**DOI:** 10.1371/journal.pone.0232604

**Published:** 2020-05-14

**Authors:** Yu Taniguchi, Shin Yamazaki, Takehiro Michikawa, Shoji F. Nakayama, Makiko Sekiyama, Hiroshi Nitta, Hidetoshi Mezawa, Mayako Saito-Abe, Masako Oda, Hiroshi Mitsubuchi, Masafumi Sanefuji, Shouichi Ohga, Nathan Mise, Akihiko Ikegami, Masayuki Shimono, Reiko Suga

**Affiliations:** 1 Japan Environment and Children’s Study Programme Office, National Institute for Environmental Studies, Ibaraki, Japan; 2 Department of Enviromental and Occupational Health, School of Medicine, Toho University, Tokyo, Japan; 3 Medical Research Center for Japan Environment and Children's Study, National Research Institute for Child Health and Development, Tokyo, Japan; 4 The South Kyushu and Okinawa Unit Center, Faculty of Life Sciences, Kumamoto University, Kumamoto, Japan; 5 Department of Neonatology, Kumamoto University Hospital, Kumamoto, Japan; 6 Research Center for Environment and Developmental Medical Sciences, Kyushu University, Fukuoka, Japan; 7 Department of Pediatrics, Graduate School of Medical Sciences, Kyushu University, Fukuoka, Japan; 8 Department of Environmental and Preventive Medicine, Jichi Medical University, Tochigi, Japan; 9 Department of ECO & CHILD subunit center, School of Medicine, University of Occupational and Environmental Health, Kitakyushu, Japan; Telethon Institute for Child Health Research, AUSTRALIA

## Abstract

**Objectives:**

No previous study has used repeated measures data to examine the associations of dog/cat ownership with wheezing and asthma prevalence among children. This prospective study used repeated measurers analysis to determine whether dog/cat ownership in childhood is an independent risk factor for wheezing and asthma, after adjustment for gestational, socio-economical, and demographical confounders confounders, in Japan.

**Methods:**

We conducted a multicenter pilot study of the Japan Environment and Children's Study (JECS) during 2009–2010. Among 440 newborn infants enrolled, 410 (52.8% males) were evaluated for dog/cat ownership in the home and history of wheezing and asthma in five follow-up questionnaire surveys (until age 6 years). Dog/cat ownership during follow-up period was categorized into four groups: 7.6% were long-term dog/cat owners, 5.9% were toddler-age owners, 5.9% were preschool-age owners, and 80.7% were never owners.

**Results:**

The prevalence of wheezing during follow-up period increased from 20.8% to 35.4% and the prevalence of asthma increased from 1.3% to 16.3%. A fitted logistic generalized estimating equation models including important confounders showed no significant associations of the interaction between dog and/or cat ownership and follow-up time with the risks of wheezing and asthma. However, the risks of wheezing and asthma were slightly lower for long-term and toddler-age dog/cat owners than for preschool-age and never owners.

**Conclusions:**

The present findings suggest that dog and cat ownership from toddler-age does not increase the risks of wheezing and asthma compared with never owners among Japanese children.

## Introduction

Epidemiological studies have reported increasing prevalence of asthma in children and young adults [1, 2], and exposure to furry pets is known as a potential risk factor for childhood allergic diseases such as asthma. Analysis of the aerodynamic characteristics of animal allergens suggests that they can be transferred in a wide range of environments [3], resulting in sensitization and asthma. Previous studies have reported beneficial [4–7] and detrimental [8–10] associations of dog and/or cat exposure with wheezing or asthma in childhood. A systematic review reported that exposure to pets slightly increased wheezing and asthma risk in older children [11]. A meta-analysis of 32 studies from the 1980s to the early 2000s found that exposure to cats had a slight preventive effect on asthma and that exposure to dogs was a risk factor [6]. Accumulated data is primarily for Western population, i.e., the United Kingdom, United States, and Sweden, in which more than 30% of households have a dog or cat [6]; however, the evidence for associations of pet and wheezing and asthma is limited in countries with lower rate of pet ownership. The Japan Pet Food Association reported that only 15% of Japanese households had a dog and that 10% had a cat [12]. Regional differences for effect of pet exposure to allergic symptom were identified [13], and the associations with wheezing and asthma should be confirmed in countries with different culture to Western country such as Japan.

Associations between dog/cat ownership and wheezing/asthma status are not always consistent throughout childhood. Collin et al investigated associations of pet ownership at six time-points with concurrent episodes of wheezing in a UK population, and reported cat ownership reduced the risk for wheezing episodes during childhood [14]. Nevertheless, to date, no investigation has been conducted to explore the associations of history of dog/cat ownership in childhood with concurrent wheezing and asthma. The present prospective study used repeated measures analysis of data (up to age 6 years) from a multicenter birth cohort study to determine if dog/cat ownership during childhood is an independent risk factor for concurrent wheezing (prodromal feature of asthma [15]) and asthma, after adjustment for socio-economical, and demographical confounders in Japan. We also conducted a stratified analysis by exposure to dog and cat separately.

## Methods

### Participants

We conducted a multicenter pilot study of the Japan Environment and Children's Study (JECS) [16, 17]. This pilot study of JECS was launched in 2009–2010 at four regional centers: Jichi Medical University, Kyushu University, University of Occupational and Environmental Health, and University of Kumamoto. In total, 440 newborn infants were enrolled in this pilot study, and self-administered questionnaires were mailed to parents/guardians every 6 months until their child reached the age of 9 years (the survey was mailed annually after age 7 years). Participants were included if data from repeated questionnaires were available on their history of dog/cat ownership and wheezing and asthma status at 6, 12, 24, 36, 54, and 72 months of follow-up. Ultimately, data from 410 out or 440 children (52.8% males) were analyzed.

The study protocol detailed in the JECS pilot study were approved by the ethics committees of Kumamoto University, Kyushu University, Jichi Medical University, University of Occupational and Environmental Health, and the National Institute for Environmental Studies. The study followed the Declaration of Helsinki. Written and oral informed consent was obtained from all parents/guardians before participation in the study.

### Dog/Cat ownership

At 12, 24, 36, 54, and 72 months, the questionnaire asked about the current situation regarding dog/cat ownership in the home. We categorized dog/cat ownership wether they experience for ownership at least once during follow-up period as owner and never owner. Moreover, we operationally defined ownership period as toddler age (12 and 24 months) and preschool age (36, 54, and 72 months) and categorized dog/cat ownership from 12 to 72 months as long-term, toddler-age, preschool-age, and never ownership. Detailed criteria and examples are shown in Table 1. We also conducted a stratified analysis by exposure to dog and cat separately and categorized ownership into four groups (long-term, toddler-age, preschool-age, and never ownership).

**Table 1:** Classification of childhood history of dog/cat ownership during follow-up.

Toddler age		Preschool age			
12 m	24 m	36 m	54 m	72 m	
YES	YES	YES	YES	YES	Long-term owners
YES	NO /NA	YES	YES	NO /NA	
NO/NA	YES	YES	NO /NA	YES	
YES	YES	NO /NA	NO /NA	NO /NA	Toddler-age owners
YES	NO /NA	NO /NA	NO /NA	NO /NA	
NO/NA	YES	NO /NA	NO /NA	NO /NA	
NO /NA	NO /NA	YES	YES	YES	Preschool-age owners
NO /NA	NO /NA	YES	YES	NO /NA	
NO /NA	NO /NA	NO /NA	NO /NA	YES	
NO /NA	NO /NA	NO /NA	NO /NA	NO /NA	Never owner

The table shows representative responses.

YES: Dog/cat present

NO: No dog/cat present

NA: No answer

### Wheezing and asthma

Although there are no standard definitions of wheezing and asthma [2], we used the criteria specified by the International Study of Asthma and Allergy in Childhood (ISAAC) [18–20]. At the 6-, 12-, 24-, 36-, and 72-month follow-up assessments, guardians were asked “Has your child had wheezing or whistling in the chest in the last 12 months (yes or no)?" [18, 21] and “Has your child had asthma during the previous 12 months (yes or no)?”. At the 6-month follow-up, the period was set as previous 6 months.

### Other variables

The covariates included baseline sociodemographic and health characteristics, namely, sex, premature birth, birth experience, cesarean section, birth weight, smoking habit in family [22], family income at 6 months of follow-up, and frequency of living room and bedroom cleaning at 12 months of follow-up.

Missing data for any of the above variables was categorized as unknown. Birth experience was categorized as present/absent sibling. Birth weight was categorized as <2500g and ≥2500g. Family income was categorized as $0–27242, $27243–45405, $45406–72647, and ≥$72648. Frequency of living room and bedroom cleaning was classified as once per month, at least once per week (but not daily), and daily.

### Statistical analysis

First, associations of baseline demographic and health characteristics with dog/cat ownership were tested by using the chi-square test. Second, we fitted the group (dog/cat ownership)-stratified logistic GEE models for the probability of wheezing and asthma, respectively, including quadratic or cubic terms for follow-up time and the first-order autoregressive correlation structure. GEE models can take into account the correlation of within-subject data. Potential confounders were evaluated for collinearity and included study area, sex, premature birth, birth experience, cesarean section, birth weight, smoking status, family income, and frequency of living room cleaning. Statistical analyses were conducted with SPSS (version 23.0; IBM Corp, Armonk, NY, USA) and SAS (version 9.4; SAS Institute, Inc., Cary, NC, USA). A *P* value of less than .05 was considered to indicate statistical significance.

## Results

### Health characteristics

During the follow-up period, 79 (19.3%) had owned a dog/cat before age 6 years and 331 (80.7%) were never owners. Among 79 dog/cat owners, 31 (7.6%) children were classified as long-term dog/cat owners, 24 (5.9%) were owners only during toddler age ("toddler-age owners"), 24 (5.9%) were owners only during preschool age ("preschool-age owners"). With respect to dog ownership, 18 (4.4%) were long-term owners, 16 (3.9%) were toddler-age owners, 11 (2.7%) were preschool-age owners, and 365 (89.0%) were never owners. With respect to cat ownership, 16 (3.9%) were long-term owners, 8 (2.0%) were toddler-age owners, 12 (2.9%) were preschool-age owners, and 374 (91.2%) were never owners. During the five follow-up assessments (median number of follow-up assessments was 4), the prevalence of wheezing increased from 20.8% to 35.4% and the prevalence of asthma increased from 1.3% to 16.3%.

Table 2 shows baseline demographic and health characteristics in relation to history of dog/cat ownership. Parents/guardians of children with a history of dog/cat ownership were more likely to have a daughter and to habitually clean the living room and less likely to have a smoking habit.

**Table 2:** Baseline demographic and health characteristics of 410 Japanese children with a history of dog/cat ownership.

	Long-term owner (n = 31, 7.6%)	Toddler-age owner (n = 24, 5.9%)	Preschool-age owner (n = 24, 5.9%)	Never owner (n = 331, 80.7%)	P-value
Sex (%)					
Male	32.3	50.0	41.7	52.3	.024
Female	58.1	41.7	58.3	42.6	
Unknown	9.7	8.3	0	5.1	
Premature birth (%)					.522
Yes	3.2	8.3	12.5	6.0	
No	96.8	91.7	87.5	94.0	
Sibling (%)					.278
Present	71.0	54.2	79.2	64.7	
Absent	29.0	45.8	20.8	35.3	
Cesarean section (%)					.129
Yes	12.9	16.7	37.5	19.9	
No	87.1	83.3	62.5	80.1	
Birth weight (%)					.123
≥2500g	90.3	87.5	79.2	84.3	
<2500g	0	4.2	20.8	10.9	
Unknown	9.7	8.3	0	4.8	
Smoking habit in family (%)					.003
Smoking near child	9.7	4.2	0	1.2	
Smoking away from child	16.1	41.7	16.7	23.6	
None	35.5	16.7	54.2	28.1	
Unknown	38.7	37.5	29.2	47.1	
Family income, USD (%)					.382
≥72,648	16.1	4.2	16.7	8.8	
45,406–72,647	16.1	16.7	25.0	19.3	
27,243–45,405	22.6	25.0	12.5	14.8	
0–27,242	6.5	16.7	12.5	6.9	
Unknown	38.7	37.5	33.3	50.2	
Frequency of living room cleaning (%)					.004
Daily	67.7	29.2	54.2	41.7	
More than once per week	25.8	62.5	37.5	53.8	
Monthly	6.5	8.3	0	1.5	
Unknown	0	0	8.3	3.0	
Frequency of bedroom cleaning (%)					.369
Daily	45.2	25.0	25.0	31.7	
More than once per week	48.4	62.5	62.5	60.4	
Monthly	6.5	12.5	4.2	4.8	
Unknown	0	0	8.3	3.0	

χ^2^ test.

### Probabilities of wheezing and asthma

**Fig 1:**
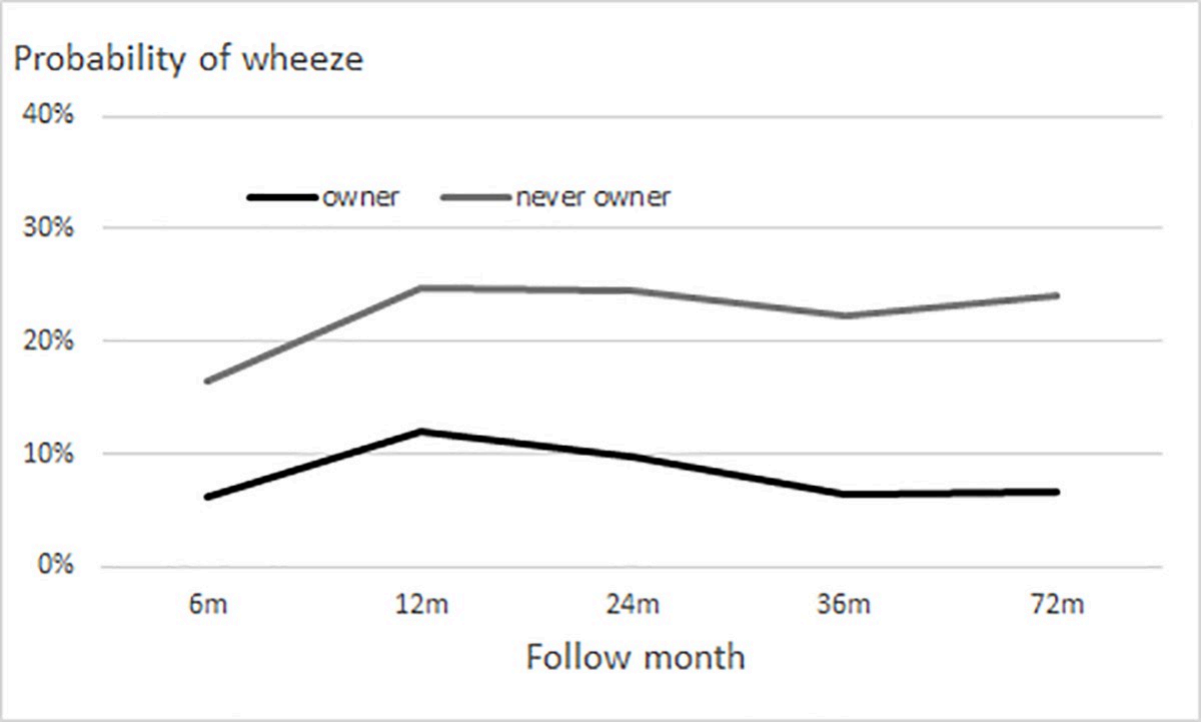
Probability of wheezing in children with a dog/cat. Group-stratified logistic generalized estimating equation models for probability of wheezing included quadratic or cubic terms for follow-up time and first-order autoregressive correlation structure (adjusted for study sex, area, and premature birth).

**Fig 2:**
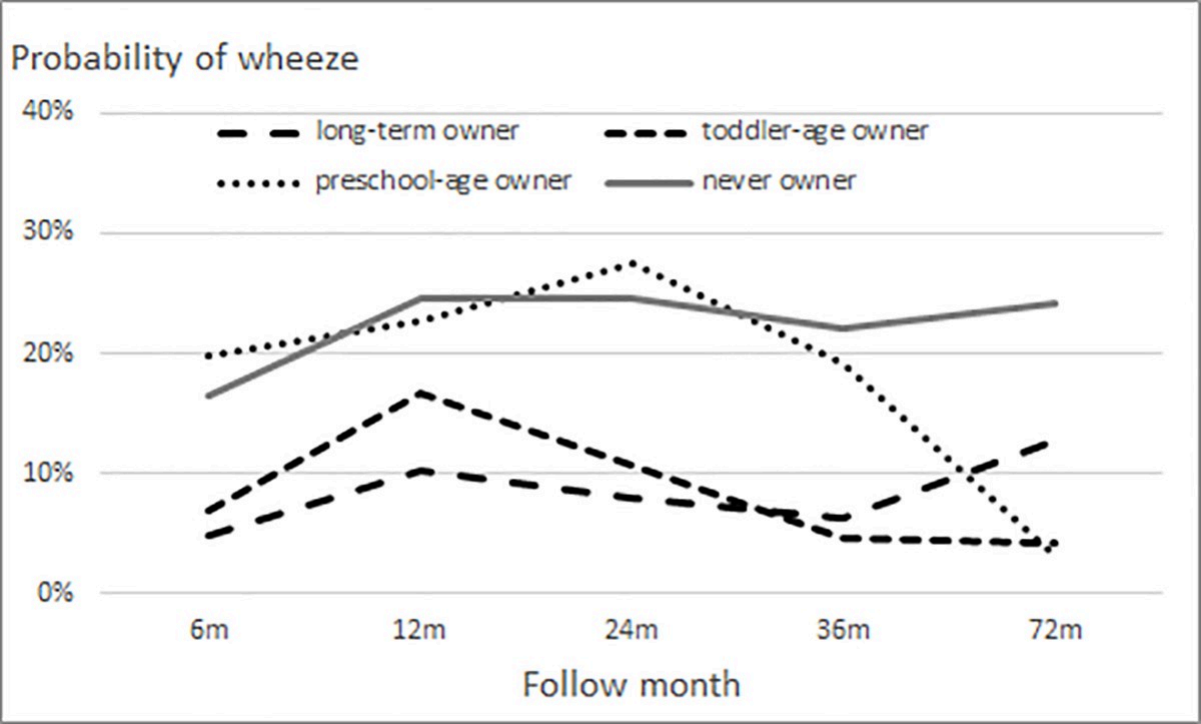
Probability of wheezing in children, by category of dog/cat ownership. Group-stratified logistic generalized estimating equation models for probability of wheezing included quadratic or cubic terms for follow-up time and first-order autoregressive correlation structure (adjusted for study sex, area, and premature birth).

Fitted logistic generalized estimating equation (GEE) models for the probability of wheezing among dog/cat owners revealed several distinct patterns. The probability during follow-up was consistently lower in owners than in never owners during childhood (Fig 1). The lowest wheezing probability in owners was 6% and the maximum probability was 12%; the corresponding values for never owners were 16% and 25%. Among dog/cat owners, long-term and toddler-age owners had slightly lower probabilities than never owners (Fig 2); however, the association of the interaction of dog/cat ownership and follow-up time with wheezing probability was not significant (P = 0.79).

**Fig 3:**
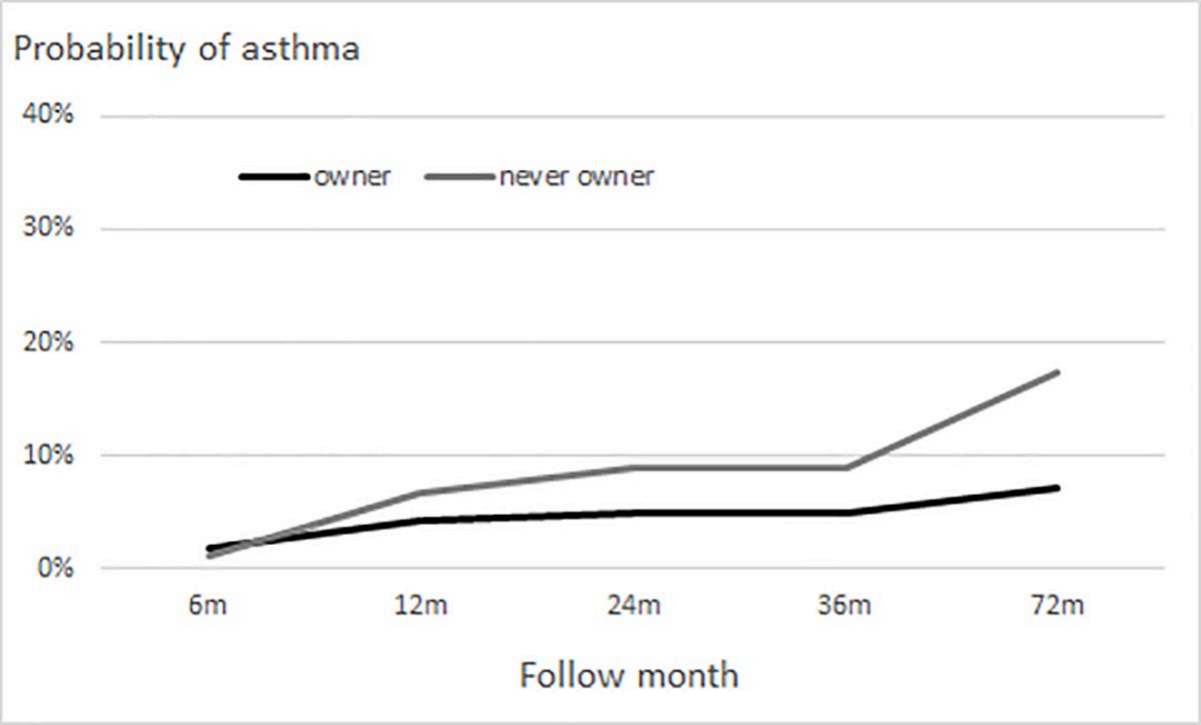
Probability of asthma in children with a dog/cat. Group-stratified logistic generalized estimating equation models for probability of wheezing included quadratic or cubic terms for follow-up time and first-order autoregressive correlation structure (adjusted for study sex, area, and premature birth).

**Fig 4:**
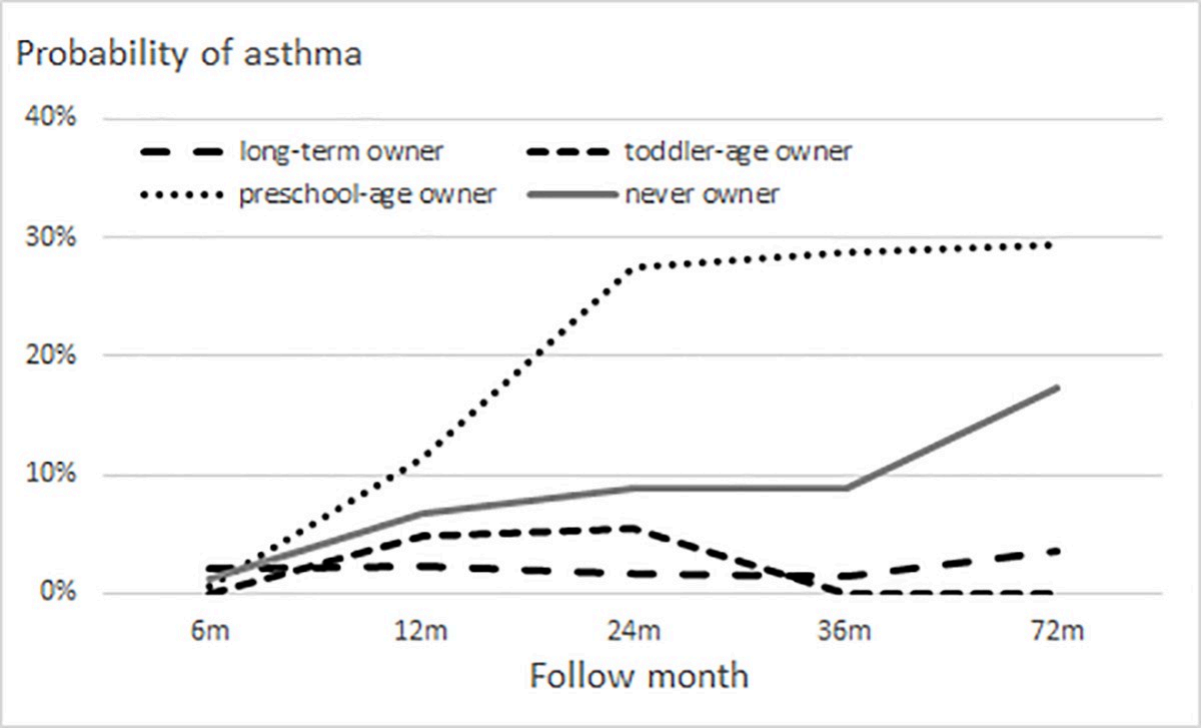
Probability of asthma in children, by category of dog/cat ownership. Group-stratified logistic generalized estimating equation models for probability of wheezing included quadratic or cubic terms for follow-up time and first-order autoregressive correlation structure (adjusted for study sex, area, and premature birth).

At the beginning of follow-up, the probability of asthma was about 1% in owners and never owners. At 12 months, the probability was slightly lower in owners than in never owners (Fig 3). The probability of asthma at 72 months was 7% in owners and 17% in never owners. Among owners, long-term and toddler-age owners had substantially lower probabilities than did preschool-age and never owners (Fig 4); the association of the interaction of dog/cat ownership and follow-up time with probability of asthma was not statistically significant (P = 0.18).

In stratified analysis, GEE models for the probability of wheezing showed no significant association of the interaction of dog ownership (P = 0.78) or cat ownership (P = 0.79) and follow-up time. The corresponding values for the probability of asthma showed no significant association of the interaction of dog ownership (P = 0.70) or cat ownership (P = 0.08) and follow-up time. We confirmed similar associations of dog and cat ownership with risk of wheezing and asthma.

## Discussion

This prospective study using repeated measures analysis of data from a multicenter birth cohort pilot study showed that growing up with dogs and cats is not an independent risk factor for wheezing and asthma, after adjustment for socio-economical, and demographical confounders. In particular, Japanese children with a history of dog/cat ownership from toddler-age had slightly lower risks for wheezing and asthma up to age 6 years.

A previous meta-analysis reported that exposure to dogs slightly increased asthma risk and that exposure to any pet during childhood was associated with higher relative risk (relative risk, 1.57; 95% confidence interval, 0.99–2.48) in studies excluding birth cohorts [6]. However, the same study found no significant associations of dog/cat ownership with asthma in four births cohorts. Collin et al reported cat ownership slightly reduced the risk for wheezing during early childhood [14]. This prospective birth cohort study in Japan stratified dog and cat owners as long-term, toddler-age (children with a history of dog/cat ownership at age 2 years old but not after age 3 years), and preschool-age owners (children with a history of dog/cat ownership after age 3 years only). As compared with never owners, long-term and toddler-age owners had consistently lower risks of wheezing and asthma until age 6 years. Our data of no significant associations of dog/cat ownership with concurrent wheezing and asthma risks are consistent with the results of previous studies conducted in Western countries.

Although we lacked information on ownership at birth for long-term and toddler-age owners, cat antigen can remain for 5 to 10 years after a cat has been removed from the environment [23]. The present findings suggest that dog/cat ownership during the first years of life is associated with slightly lower risks of wheezing and asthma, as compared with never owners and ownership after age 3 years. Some evidence suggests that two mechanisms explain why children with a history of dog/cat ownership from toddler-age tend to have slightly lower risks of wheezing and asthma. First, dog/cat ownership may increase the diversity and richness of the environment biome, as exposure to pets appears to change the microbiome profile [24, 25] in early childhood. Previous studies revealed that pet keeping such as dog and cat is related to microbial alternations [26–31]. Composition of human gut microbiome might play a key role in decreasing of allergies in childhood. Second, early exposure to bacterial endotoxins may protect against allergy development. Dog/cat ownership is strongly associated with indoor levels of bacterial endotoxins [32]. Bacterial endotoxin is known to induce production of Th1 associated cytokines, interferon-γ, and interleukin-12, and to prevent IgE production. Furthermore, bacterial endotoxin protects against allergy by inducing production of A20 in lung epithelial cells [33]. The mechanism underlying the associations of dog/cat ownership in childhood with wheezing/asthma risk is not determined in the present study, but our data supports the hygiene hypothesis [34]. It is likely that early life dog/cat exposures stimulate Th1 cell and may contribute the delicate balance for Th1/Th2 ratio. The findings suggest that dog and cat ownership from toddler-age does not increase the risks of wheezing and asthma among Japanese.

This study has strengths that warrant mention. First, the present repeated measures data were collected at five time points (12, 24, 36, 54, and 72 months) during the follow-up period, which enabled analysis of long-term, toddler-age, preschool-age, and never dog/cat ownership. Second, the present study included time series analysis of the risks of wheezing and asthma until age 6 years. Third, sociodemographic and health characteristics were analyzed, which allowed us to examine independent associations of dog/cat ownership in childhood with wheezing and asthma, after controlling for socio-economical, and demographical confounders. Forth, the percentage of homes with dogs/cats is completely different between Japan and Western countries [35]. The present data from a county with low rates of pet ownership is beneficial, because the relationships to pet animals might differ by culture.

This study has some limitations. First, the reason why preschool-age owners showed obviously lower probability of wheezing at age 6 years is not clear at present, however it is possible that data instability due to the stratified analysis for dog/cat ownership contributed to this finding. Second, although we propose two mechanisms to explain why children with a history of dog/cat ownership from toddler-age tend to have consistently lower risks of wheezing and asthma up to age 6 years, this study did not identify the clear pathway. The hygiene hypothesis proposes that childhood exposure to germs develops the immune system. Future studies should attempt to identify the mechanism responsible by examining bio-specimen sample, including microbiome, endotoxin level, Th1/Th2 balance, and allergic sensitization such as specific IgE antibodies [36] and skin prick testing among dog/cat owners in toddler-age. Third, family inheritance may be a confounder of the association between pet ownership and asthma [6]. Allergic parents may actively avoid pets [37, 38]. Eller et al examined inconvenient ground access, in addition to family history of allergy, and found that both variables reduced the probability of cat ownership [38]. Although the present study could not examine substantial social and housing factors as potential confounders in all participants, we conducted a preliminary analysis of participants with the necessary data (data were available for 37.0% of mothers and 33.4% of fathers). After adjustment for maternal and paternal history of allergy, we confirmed similar results for the associations of dog/cat ownership in childhood with wheezing and asthma risk (data not shown). Children with dogs had less asthma at age six after adjustment for potential allergy medication [7], which could explain the remaining association after controlling for potential allergy.
